# Hemichorea-Hemiballism as a Manifestation of Hyperglycemia

**DOI:** 10.7759/cureus.19330

**Published:** 2021-11-07

**Authors:** Maria Maia, Ana Patrícia Moreira, Ana Isabel Gonçalves, João Espírito Santo, José Araújo

**Affiliations:** 1 Internal Medicine, Hospital Beatriz Ângelo, Lisboa, PRT; 2 Neurology, Hospital Beatriz Ângelo, Lisboa, PRT

**Keywords:** hemiballismus, neuroleptics, hyperglycemia, diabetes, chorea

## Abstract

Hemichorea-hemiballism associated with hyperglycemia is a syndrome characterized by a sudden occurrence of hemichorea, or its more severe expression hemiballism, in patients with non-ketotic hyperglycemia. Hemichorea-hemiballism tends to occur more commonly among elderly people and women of Asian origin. The authors present two rare cases of patients who manifested choreiform and ballistic movements of the limbs and concomitant non-ketotic hyperglycemia. Radiological findings were congruent with hyperglycemia etiology. These cases show that it is important to be aware of hemichorea-hemiballism associated with hyperglycemia, as there is a possible treatment and, if detected early on, a direct impact on prognosis.

## Introduction

Hemichorea-hemiballism associated with hyperglycemia (HHAH) was first described in 1960. However, no more than 200 cases have been reported ever since [1]. HHAH is characterized by the sudden occurrence of hemichorea, or its more severe expression hemiballism, in patients with non-ketotic hyperglycemia [2]. Non-ketotic hyperglycemia is the second most frequent cause of the hemichorea-hemiballism syndrome, only after vascular origin [3,4]. The typical triad of HHAH consists of involuntary movements, striatal abnormalities on neuroimaging, and non-ketotic hyperglycemia with known or previous unrecognized diabetes [1,4]. Usually, HHAH occurs unilaterally, but in some cases, it can occur bilaterally [5]. HHAH is more common among elderly people, especially women of Asian origin [6]. Age seems to be the greatest risk factor for HHAH.

HHAH is characterized by a severe increase in blood sugar levels and negative ketones in the urine. Type 2 and less frequently type 1 diabetes are associated with HHAH. Diabetes was newly diagnosed in several cases of HHAH [1]. Laboratory findings in reviewed literature include a series of 53 patients with HHAH from Oh et al. with mean serum glucose level upon admission of 481.5 mg/dL (ranging from 169 to 1264 mg/dL) and mean glycated hemoglobin (HbA1c) of 14.4% (ranging from 9.9% to 19.2%) [6]. Other studies and series have confirmed these average serum glucose values [5].

Characteristic radiographic findings of HHAH are hyperdensity changes in the contralateral striatum in computed tomography (CT) scan and high signal changes in the contralateral striatum in T1-weighted magnetic resonance imaging (MRI) and equal or low signal in T2-weighted MRI. Additionally, MRI plays an important role in etiological investigation [7,8]. The lesions typically resolve over time, with eventual normalization of the CT scan or MRI.

The main treatment of HHAH is the normalization of blood glucose. Hemichorea-hemiballism slowly improves in the days after serum glucose correction [1,6,9]. In addition, neuroleptics are often used to expedite symptomatic resolution, as the symptoms of HHAH can cause discomfort to the patients and, in extreme cases, lead to rhabdomyolysis and renal failure. In some series, different combinations of glycemia control, neuroleptics, dopamine-depleting agents, benzodiazepines, and anticonvulsants were used [1,9].

The authors present two clinical cases of this rare pathophysiological process associated with neurological manifestations of hyperglycemia.

This article was previously presented as a poster at the 2019 European Congress of Internal Medicine on August 30, 2019, in Lisbon, Portugal.

## Case presentation

**Figure 1:**
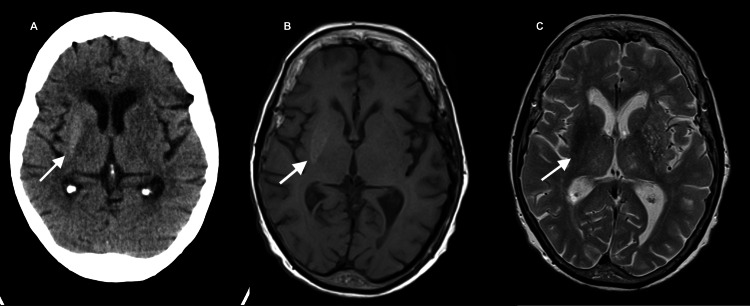
Image study of case 1. A. Head CT scan showing lenticular and right capsular hyperdensity. B. Head MRI, T1, showing hypersignal in the lenticular right nucleus. C. Head MRI, T2, showing hyposignal of the lenticular right nucleus.

The first case concerns a 91-year-old female patient of Caucasian origin. The patient had a previous history of type 2 diabetes treated with linagliptin and auricular fibrillation under apixaban. She presented to the emergency department with a four-day history of involuntary movements of the left arm and foot. The neurological exam showed a Glasgow Coma Scale of 15, with choreiform movements (random, low amplitude, flowing movements) of the left arm, leg, and foot, more proximal. These movements were recurrent and caused pain and discomfort to the patient. On laboratory investigation, the patient’s serum glucose was 460 mg/dL (HbA1c 14%), there was no acidosis, and urinalysis was negative for ketone. CT scan revealed no ischemic or hemorrhagic lesion, but a lenticular and right capsular hyperdensity was noted (Figure 1A). MRI showed a change in the signal of the lenticular right nucleus, with hypersignal in T1 (Figure 1B) and hyposignal in T2 and fluid-attenuated inversion recovery (FLAIR) imaging (Figure 1C).

Overall, the clinical and radiological examination was consistent with HHAH. After hydration and insulin, risperidone 0.5 mg twice a day was started. Four days later, there was a significant improvement in the symptoms. The patient was discharged on a scheme of basal insulin, metformin, linagliptin, and risperidone. Two weeks later, the patient was asymptomatic, with glycemia within target levels, leading to the suspension of risperidone. An MRI was done six months after the event, showing the total resolution of the lesions previously identified.

**Figure 2:**
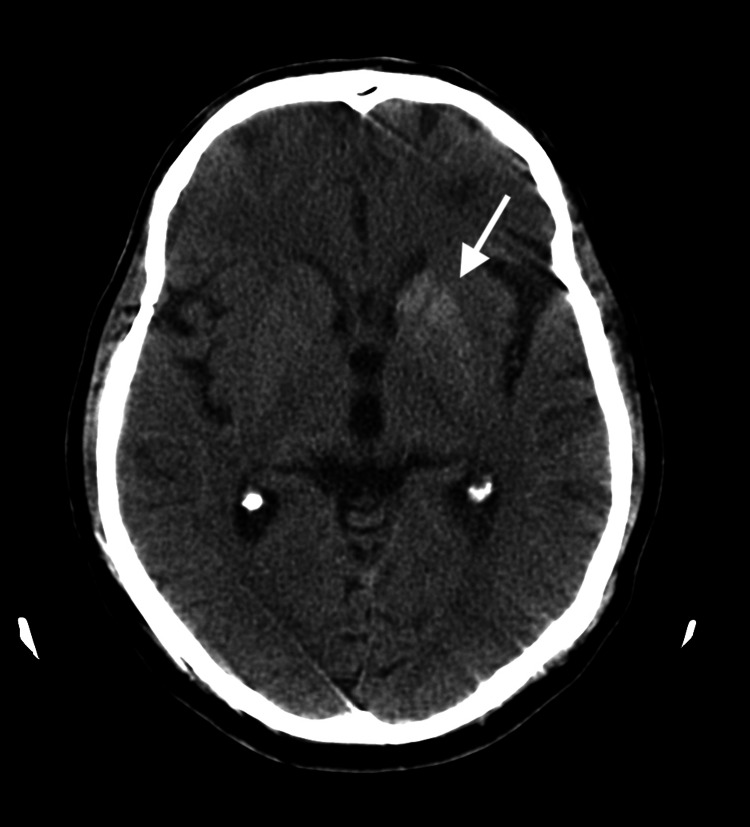
Head CT scan of case 2 showing a hyperdensity of the left globus pallidus.

The second case regards a 60-year-old male patient of Caucasian origin. The patient suffered from arterial hypertension, dyslipidemia, and insulin-treated type 2 diabetes, complicated by nephropathy. The patient presented to the emergency department complaining of involuntary movements of the right hemibody that had been increasing over a month. Neurological examination showed proximal and distal flinging movements of low and large amplitude (ballistic and choreiform movement) in the right arm and leg. Laboratory investigation revealed a glycemia level of 350 mg/dL (HbA1c 12%), with a negative ketone test and no acidosis. CT scan showed a hyperdensity on the left globus pallidus (Figure 2).

Hydration and insulin were started for the patient and then titration of haloperidol (1 mg three times a day), valproate (500 mg twice a day), and lorazepam (1 mg a day) was commenced. There was a slight improvement in the patient's condition and the patient was discharged with residual minor chorea, under an adjusted scheme of basal-bolus insulin and linagliptin for diabetes, and haloperidol for hemichorea. After discharge, the symptoms slowly improved, with complete resolution after two months. During follow-up, glycemia was controlled and haloperidol was tapered off with no recurrence of the abnormal movements. After eight months, an MRI showed complete resolution of the lesions.

## Discussion

HHAH is a syndrome characterized by a sudden occurrence of hemichorea, or its more severe expression hemiballism, in patients with non-ketotic hyperglycemia [2]. The most common manifestation of this syndrome is the acute or subacute onset of involuntary limb activity. Less common are involuntary movements of facial muscles, namely, the jaws and tongue [8]. Usually, these involuntary movements are unilateral, which was the case of the two patients discussed in this article. While there are some reported cases of bilateral movements, such cases seem to represent only 10% of the HHAH cases [6]. The involuntary movements often get worse daily and can be classified from mild chorea to severe ballism based on their type and severity. In the second case presented, the involuntary movements were severe and had worsened over a considerable period. The time gap between the beginning of the symptoms and the first medical evaluation could be one of the reasons for the slower resolution of the syndrome, as seen in other cases with longer evolution [10,11]. However, it is difficult to reach conclusions, as other factors can be involved.

In both cases, the patients were of Caucasian origin. One of the patients was a 91-year-old woman, following data tendency from previously recognized HHAH patients. However, even if female sex seems to be a risk factor, the literature also presents some cases of male patients. A case series of 53 patients revealed a female-to-male ratio of 1.7:1, with an average age of 71.1 years and 91% of the patients were of Asian origin [6]. In another series of 20 cases from 2016 in Peru, a female-to-male ratio of 1.3:1 was found, with an average age of 67.8 years [1]. The majority of the literature has its origin in Asian countries, but cases all over the world have been reported [1].

In the majority of cases, it is difficult to determine for how long the serum glucose values were elevated. In these cases, the value of HbA1c can be an asset in confirming the diagnosis and guiding therapeutics. Even though the two patients presented were already diagnosed, it was the HbA1c level that showed that serum glucose values were high above the target the majority of the time.

Concerning the treatment, the most effective drug classes block postsynaptic dopamine D2 receptors. Typical antipsychotics like haloperidol or perphenazine were most commonly used in the past three decades [2,9]. Atypical antipsychotics, because of their more secure profile, have also been used in some cases with proven benefits [9]. Lately, dopamine-depleting agents, like tetrabenazine, have been reported to show improvement in hemiballismus due to stroke and non-ketotic hyperglycemia [2].

As it was described, in the first case, there was a rapid evolution of symptoms after blood sugar control and the use of risperidone at a small dosage. Risperidone was the choice due to its safety profile and the shorter-time evolution of the symptoms. Simply controlling the glycemia could lead to symptom resolution, but the discomfort of the patient with the abnormal movements was the reason to start immediate treatment with an antipsychotic. Even if it is not the most common choice, there are other cases in the literature where this choice was made and the results were also favorable [9].

The second case was a bigger challenge as the symptoms were more severe and did not improve in the first days. In this case, there was a need to combine different classes of medication, besides neuroleptics, to better control the symptoms, with extra care in controlling the blood glucose level. The persistence of symptoms despite all treatments could also reflect permanent damage. Deep brain stimulation of the internal globus pallidus has been suggested as an alternative treatment in the cases where the symptoms persevere [9,10]. The second patient was followed up closely in case other interventions would be needed.

Despite different treatments, both patients showed complete resolution of the abnormal movements, and imaging findings were also reversible. There was no recurrence of the symptoms in these patients. Literature references some atypical cases, with unremitting severe movements and late recurrence, usually associated with hyperglycemia leading to the need of continuing a strict glycemic control [12].

## Conclusions

HHAH is a rare syndrome. However, if detected early on, glycemic control and neuroleptics can solve HHAH in a short period. Therefore, clinicians should have a higher index of suspicion regarding this syndrome, especially in poorly controlled diabetic patients, because early diagnosis, intervention, and metabolic control are key to effective treatment of HHAH.
